# Sleep contributes to preference for novel food odours in *Drosophila melanogaster*

**DOI:** 10.1038/s41598-021-88967-1

**Published:** 2021-04-30

**Authors:** Fuminori Tanizawa, Hiroyuki Takemoto

**Affiliations:** 1 Future Scientists’ School, Shizuoka University, Oya 836, Shizuoka, 422-8529 Japan; 2 Research Institute of Green Science and Technology, Shizuoka University, Oya 836, Shizuoka, 422-8529 Japan

**Keywords:** Behavioural ecology, Ecophysiology

## Abstract

The importance of sleep in maintaining cognitive functions such as learning and memory has been reported in both vertebrates and invertebrates. Previous studies demonstrated that sleep deprivation impaired the olfactory memory retention of fruit flies as described in the classical conditioning paradigm. Here, we show that sleep deprivation leads to a preference for the odours of the rearing environment in *Drosophila melanogaster*. Flies whose sleep had been disturbed with periodic rotation stimuli during night-time preferred apple cider vinegar (ACV) to broth, while this preference was lower in flies without sleep deprivation and those rotated during daytime. Experiments using single odours showed an increase in responses to ACV due to sleep deprivation. These results suggest that sleep functions in food odour preference. Flies grown on medium supplemented with ACV showed greater preference for ACV, and those grown with broth supplementation showed a greater preference for broth under sleep-deprived conditions. These results suggest that flies with night-time sleep deprivation become attached to the environment on which they have developed, and that sleep contributes to preference for novel food odours. This study offers an approach to investigating the interaction between sleep and neural disorders concerning cognitive deficits towards novel stimuli.

## Introduction

Sleep is a widespread phenomenon in animals^1^. The function of sleep is largely unknown, but it involves the maintenance of cognitive abilities^2,3^. Many studies have suggested that sleep enhances memory acquisition, consolidation, and integration in various learning tasks^4–6^. Learning and memory is the fundamental process involved in a wide range of behaviours such as foraging, social interactions, mating, and danger avoidance in animals^7^. Therefore, sleep deficits can cause behavioural changes leading to important ecological consequences in each animal species.

Sleep in animals is defined by behavioural criteria: consolidated circadian periods of immobility, a species-specific posture, elevated sensory thresholds, and homeostatic regulation^8,9^. In *Drosophila melanogaster*, night-time circadian immobility periods with a preferred posture and location were observed^8,10^ using a standard locomotor assay^11^ and video recordings. Since then, *D. melanogaster* has been used as a model animal in sleep research^12–14^*.* Previous studies have demonstrated that sleep deprivation with periodic mechanical stimuli impairs memory consolidation and retrieval in olfaction^15–17^. While many studies suggest the importance of sleep, sleep loss does not necessarily result in a lethal physiological impact on the survival of individual *D. melanogaster*^18^*.* To understand the function of sleep in *Drosophila*, its ecological significance needs to be investigated, since the behaviour of individuals in their environment is also important for the survival and reproduction of the species^19,20^. This present study aims to find the effect of sleep deprivation on behaviour in an ecological context in *Drosophila* to better understand the function of sleep in animals.

Fruit flies use a wide variety of host materials, although they are mainly saprophytic and preferentially use rotting plant materials as hosts in the natural environment^21^. Flies show olfactory responses to a broad range of chemicals and food materials, not only those originating from plants, such as apple cider vinegar (ACV), but also those from animals such as fish extracts (broth)^22,23^. However, the preference among those odours has been less investigated, and there are no reports about the effect of sleep deprivation on olfactory preference in *D. melanogaster*. In this study, we first tested a hypothesis that sleep deprivation in *D. melanogaster* alters their food odour preferences. We counted the number of flies subjected to different rotation schedules (no rotation, night-time rotation, or daytime rotation) attracted by odours of distinctive food sources, apple cider vinegar (ACV) and broth in a trap assay, when the odours were supplied in direct choice tests or individually using water as a control. For the validation of sleep deprivation, locomotor activities of flies were measured in the standard locomotor assay. The odour sources were determined based on their different origins, ACV from plant materials and broth from animal materials, so that they were discriminated as different sources by flies.

To further understand the ecological significance of sleep, we focused on the odour of the rearing environment. Many animals are likely to prefer the environment in which they developed^24–26^ and acquire preference for odours of the natal environment through their experiences in their developmental sites^27–30^. In fruit flies, the experience of a particular food during the larval stage did not affect adult preference for the food, but that during the adult stage increased the acceptability of the food experienced^31^. However, the odour experienced during the larval stage may be involved in memory consolidation in the later adult stage^32^. In this study, we hypothesized that the odour of the rearing environment affects changes in olfactory preference in sleep-deprived flies. We reared flies on medium supplemented with ACV or broth and then tested the preference between ACV and broth in flies who were grown on each medium (ACV medium or broth medium) under different sleep conditions (no rotation or night-time rotation).

## Results

### Locomotor activity

**Figure 1:**
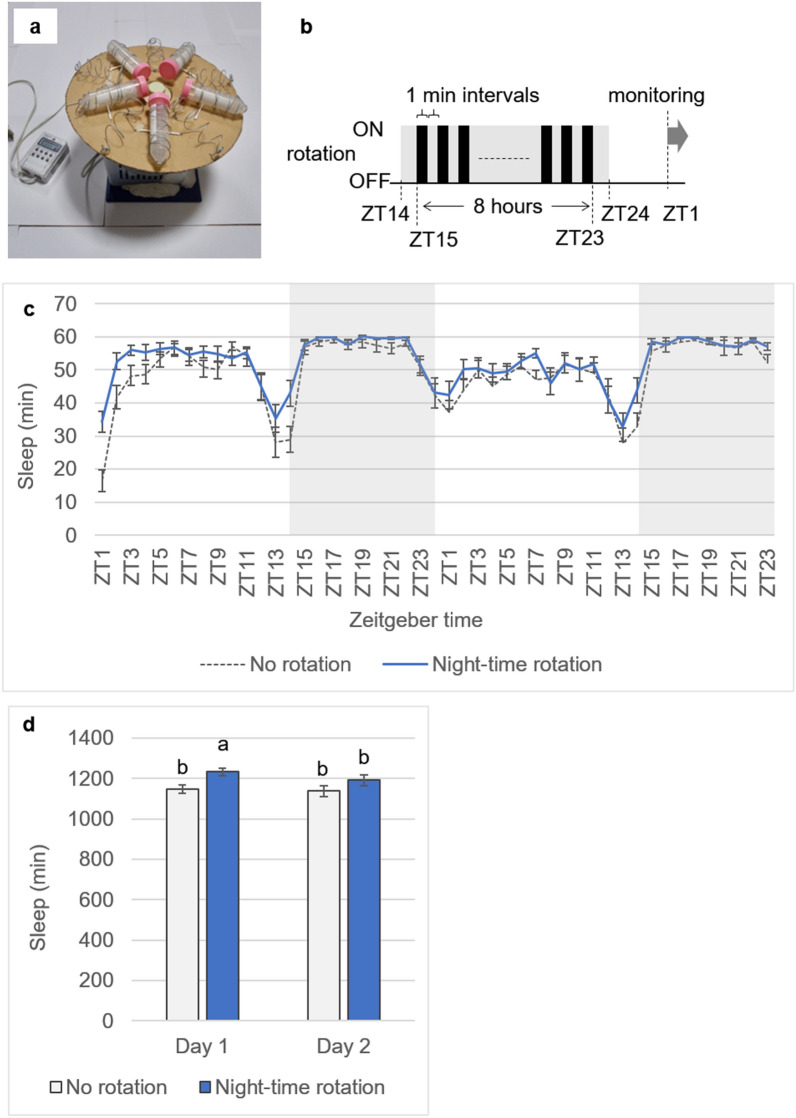
Sleep deprivation treatments with rotation treatments. (**a**) Custom-made centrifuge device used to provide periodic mechanical stimuli for sleep deprivation. A horizontal fibreboard disc (30 cm diameter) was installed on a fan motor. The power was controlled by a digital timer relay. (**b**) Time schedule of rotation treatments. The centrifuge device was turned on and off every minute for 8 h. Night-time rotated flies experienced the periodic mechanical stimuli from ZT15 to ZT23. The activity monitoring was started from ZT01 on the next morning. (**c**) Sleep time of flies with night-time rotation (N = 21) or without rotation (N = 22) in 1-h bins measured using the standard locomotor activity assay. Sleep rebound was found especially on the next light period following the rotation treatment. (**d**) Total sleep time in each day. Night-time rotated flies showed ca. 7.5% longer sleep than no rotation flies on day 1. Different letters indicate significant differences (α = 0.05; GLMM with Holm–Bonferroni correction).

In order to evaluate the effect of rotation on sleep of fruit flies, locomotor activities of flies were monitored using the standard locomotor activity assay with single infrared beam interruptions^8,10,33,34^. Periodic mechanical stimuli were provided to flies using a custom-made centrifuge device (Fig. 1a) for 8 h with 1 min on and off intervals (Fig. 1b). Sleep time of flies with rotation in night-time or those without rotation were shown in 1-h bins (Fig. 1c) and in total amount in each day (Fig. 1d). Sleep rebound was found especially on the next light period following the rotation treatment. Night-time rotated flies showed ca. 7.5% longer sleep than no rotation flies on day 1. The effect of rotation treatment on total sleep time was different due to the monitoring days, as an interaction was found between those explanatory variables (GLMM; δdeviance = 4.4096, *P* = 0.032) in the analysis of the whole statistical model with both rotation and monitoring day were included as explanatory variables. Further interpretation of effects of the explanatory variables on total sleep time were made using separate models on each explanatory variable (rotation treatment or monitoring day), because interpretation of the effect of variables cannot be done separately when there is an interaction between the two variables^35^. The sleep time of flies with night-time rotation was significantly longer than those without rotation on the day 1 (GLMM; δdeviance = 7.8345, *P*_*adj*_ = 0.030), and the increased sleep time in flies with night-time rotation on the day 1 was decreased on the day 2 (GLMM; δdeviance = 15.0639, *P*_*adj*_ = 0.006) and restored as the difference due to the rotation was not found in day 2 (GLMM; δdeviance = 1.8715, *P*_*adj*_ = 0.417). No significant difference was found between sleep times of the monitoring day 1 and day 2 in flies without rotation (GLMM; δdeviance = 0.8033, *P*_*adj*_ = 0.364). In addition, no significant difference was found between the total sleep times of no rotation flies in day 1 and that of night-time rotated flies in day 2 (GLMM; δdeviance = 1.3811, *P*_*adj*_ = 0.432), and the total sleep time of night-time rotated flies in day 1 was significantly longer than that of no rotation flies in day 2 (GLMM; δdeviance = 8.0843, *P*_*adj*_ = 0.006) (Fig. 1d, Supplementary Information).

### Olfactory response (trap assay)

**Figure 2:**
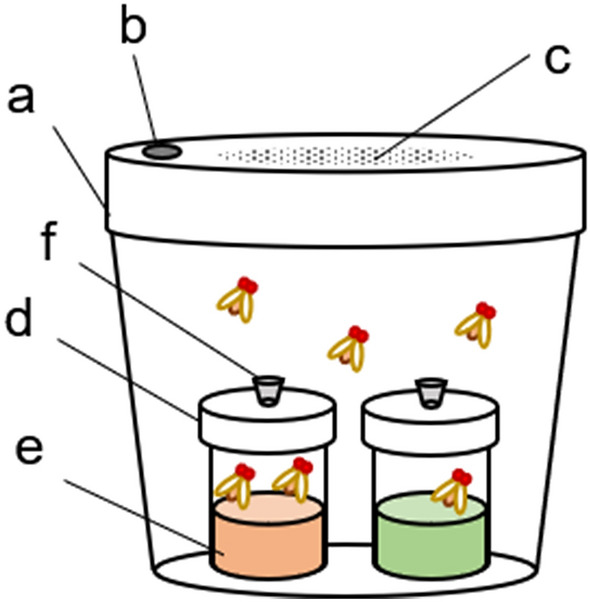
Test apparatus for olfactory response tests (trap assays). (**a**) 500 ml plastic container. (**b**) Opening (8 mm in diameter) for the rapid transfer of flies. (**c**) Fifty tiny ventilation holes. (**d**) Glass vial. (**e**) Odour source sample. (**f**) Tube made of a pipette tip inserted into a hole (2 mm in diameter).

**Figure 3:**
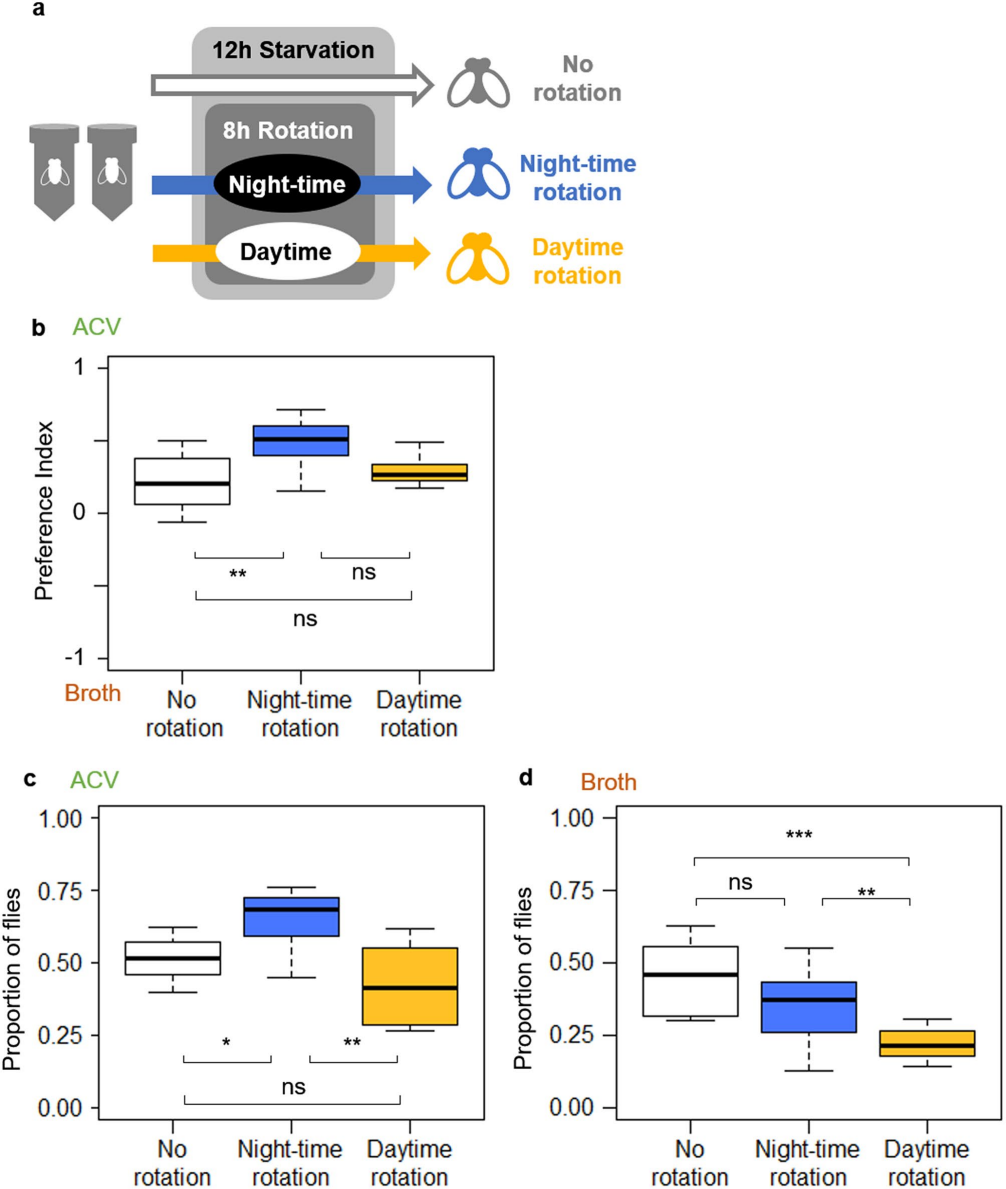
Olfactory responses of fruit flies under different rotation schedules for apple cider vinegar (ACV) or broth. (**a**) Treatments of flies with different rotation schedules (**b**) Olfactory preferences of flies with different rotation schedules between ACV and broth shown in a preference index (1 = absolute preference for ACV and − 1 = absolute preference for broth) (N = 10). **P_adj_ < 0.01, ns, not significant (GLMM with Holm–Bonferroni correction). (**c**) Response rates of flies under different rotation schedules to ACV, and those to broth (**d**). The proportion of flies trapped within the odour source vial among live flies was shown in boxplots (*N* = 10). *P_adj_ < 0.05, **P_adj_ < 0.01, ***P_adj_ < 0.001, ns, not significant (GLMM with Holm–Bonferroni correction).

Olfactory preference was determined based on the methods of a previous study with modifications^23^ using an olfactory test apparatus (Fig. 2). We prepared three treatments of flies with different time schedules of rotation treatments; i.e. no rotation, night-time rotation and daytime rotation (Fig. 3a).

### Experiment 1: Preference for Apple cider vinegar (ACV) or broth

The olfactory preferences of the flies for ACV or broth was different due to rotation treatments (GLMM; δdeviance = 11.4331, *P* = 0.001) (Fig. 3b). Flies with night-time rotation showed a greater preference for ACV over broth than flies without rotation (GLMM; δdeviance = 8.9866, *P*_*adj*_ = 0.009). No significant differences were found between the preferences of flies with night-time rotation and those with daytime rotation (GLMM; δdeviance = 2.5443, *P*_*adj*_ = 0.092) or between flies without rotation and those with daytime rotation (GLMM; δdeviance = 4.3489, *P*_*adj*_ = 0.058).

### Experiment 2: Response rate to each odour source

The olfactory responses to ACV changed with rotation treatments of flies (GLMM; δdeviance = 15.7057, *P* < 0.001) (Fig. 3c). Flies with night-time rotation showed greater response to ACV than flies without rotation (GLMM; δdeviance = 4.6933, *P*_*adj*_ = 0.040) and flies with daytime rotation (GLMM; δdeviance = 13.2358, *P*_*adj*_ = 0.003). No significant difference was found between flies without rotation and those with daytime rotation (GLMM; δdeviance = 3.4432, *P*_*adj*_ = 0.062).

The olfactory responses to broth changed with the rotation treatments of the flies (GLMM; δdeviance = 19.0801, *P* < 0.001) (Fig. 3d). Flies with daytime rotation showed less response to broth than flies without rotation (GLMM; δdeviance = 18.9513, *P*_*adj*_ < 0.001) and flies with night-time rotation (GLMM; δdeviance = 8.1299, *P*_*adj*_ = 0.006). No significant difference was found between flies with night-time rotation and those without rotation (GLMM; δdeviance = 3.2401, *P*_*adj*_ = 0.054).

### Experiment 3: Effect of the rearing environment of the preference

**Figure 4:**
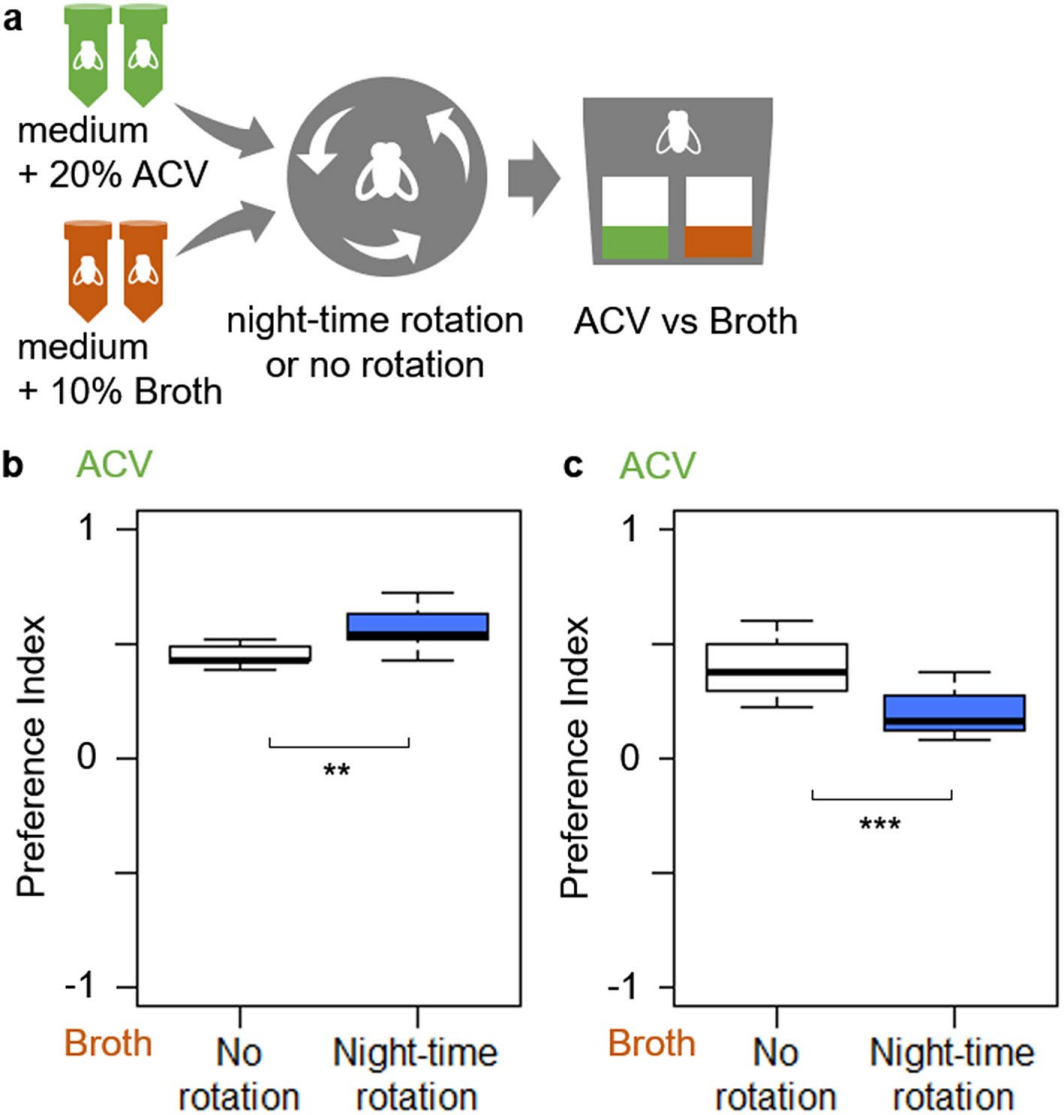
Olfactory preference of fruit flies under different sleep conditions for ACV and broth. (**a**) overview of the experiment using fruit flies reared on mediums supplemented by ACV or broth (**b**) Olfactory preferences of flies reared on ACV medium shown in a preference index (1 = absolute preference for ACV and − 1 = absolute preference for broth) (N = 10). (**c**) Olfactory preferences of flies reared on broth medium (N = 10). **P < 0.01, ***P < 0.001 (GLMM).

The effect of rotation treatment was different due to media on which flies had been grown (ACV medium or broth medium; Fig. 4a), as an interaction was found between the two explanatory variables (rotation and rearing environment) (GLMM; δdeviance = 16.8574, *P* < 0.001) in the analysis of the whole model including the effect of rotation and rearing environment as explanatory variables. Further analysis and inference were made by the separate models on each rearing environment (ACV medium and broth medium). Flies on ACV medium with night-time rotation showed a greater preference for ACV over broth than flies without rotation (GLMM; δdeviance = 7.6648, *P* = 0.004) (Fig. 4b). On the other hand, flies on broth medium with rotation showed a greater preference for broth than flies without rotation (GLMM; δdeviance = 12.5141, *P* < 0.001) (Fig. 4c).

## Discussion

The temporal increase of sleep time in flies with night-time rotation was regarded as sleep rebound due to sleep deficit^36^, which suggested that the sleep of flies was deprived with the rotation treatment. The olfactory preference of flies was altered by the sleep deprivation treatments. Flies whose sleep was disturbed by night-time rotation preferred ACV to broth. The change in preference was attributed to an increased response to ACV as the response to ACV in the no-choice experiment was increased in the night-time rotated flies. The change in preference in the night-time rotated flies reflected a greater preference for the odour of the rearing environment. The night-time rotated flies grown on medium supplemented with ACV showed a greater preference for ACV, and flies grown on medium supplemented with broth showed a greater preference for broth.

These results indicate that sleep has some functions in foraging behaviour in fruit flies. In addition, the response rate to broth in the no-choice experiment was decreased in daytime-rotated flies. This suggests that the effect of sleep deprivation was unique to the time period. The circadian periods of sleep in *Drosophila* more frequently take place at night^8,37^. In this study, night-time rotation disrupted longer time of sleep, and daytime rotation was the way around. However, the effect of the daytime rotation was not simply weaker than that of night-time rotation but qualitatively different. The different amounts of sleep deprivations may cause different behavioural changes. The reversal effects due to amount of sleep deprivation was observed in humans, as short time sleep deprivation (24–48 h) reduced interest in high-risk sensational activities that require the expenditure of energy, while longer-term sleep deprivation (75 h) may lead to a resurgence of that interest the other way around^38^. To investigate further detailed physiological and cognitive processes involved in the change in preference, correlations between the amount of sleep deprivation and preference are needed to be measured at the level of individual flies.

To investigate the ecological significance of the change in olfactory preference, we tested the hypothesis, which stated that the odour of the rearing environment affects the change in olfactory preference in sleep-deprived flies. If so, the increased preference for ACV in sleep-deprived flies in the former experiments might be due to a generalization of propionic acid regularly supplemented in the standard media as an antimicrobial agent. The hypothesis was supported; sleep-deprived flies showed greater preferences for odours related to the supplements given in the rearing environment. The host materials preferred by fruit flies in the natural environment are rotting plant materials^21^. Rotting plants emit volatile organic compounds such as alcohols, acids and esters^39^. These compounds that are associated with rotting plants generally attract fruit flies in trap tests^23^. In the current study, the materials preferred by sleep-deprived flies were reversed with previous exposure via media, even though broth was a more atypical host material than ACV (which is reminiscent of rotting apples). The results suggest that responses to previously experienced odours are increased in sleep-deprived flies, while the other less preferred odour may be associated with novel materials that the flies have never experienced. Thus, from the viewpoint of ecological function, sleep contributes to preference for novel food odours.

An enhanced preference for odours of the rearing environment would benefit sleep-deprived flies by increasing the possibility of their finding a location without the time of exploration and allocate the time to have a longer period of sleep. Sleep-deprived flies exhibit longer periods of compensation sleep (sleep rebound) than normal flies^8,10^. Flies are expected to face a trade-off in terms of allocating their time between sleeping and carrying out daily activities, including foraging^19^. Although searching for novel host material would be beneficial to flies, it requires energy and exposure to the risk of predation^40^. The results suggested that flies did not search for novel host material as their preference for novel odours decreased. This is also reasonable because physical stress can decrease risk-taking behaviour and exploration in animals^41,42^. In humans, sleep deprivation reduces self-reported risk-taking propensity^43–45^. On the other hand, the increased preference for familiar odour may result in attachment to the developmental site. However, staying in the developmental site is also risky because the quality of the materials on which the flies developed may no longer be available since the materials may have rotten. It is more likely that flies rapidly locate a site to rest using olfactory cues with high detectability. Under sleep deprivation, flies may exhibit cognitive processes that are dependent on heuristic decision making^46^.

Since the assay of olfactory responses in this study was performed using group of flies, the choices of flies in the current study might include both individual and collective decisions. *Drosophila* flies aggregate on a food patch where social interactions such as mating take place^47^. Previous studies showed that olfactory responses of *D. melanogaster* flies can be affected by social interactions from other individuals. The male-produced lipid pheromone 11-cis-vaccenyl acetate acts as an aggregation pheromone for males and females^48^. The landing rate of female flies to food materials was increased by housing of males with the materials for 24 h^49^. Female fruit flies prefer to oviposit on media patches that had been previously housed by ovipositing females of the same species^50^. In the current study, those effects of social interactions were not eliminated, although some conditions might be insufficient for holding the social interactions. Firstly, the duration of trap assay (16 h) was shorter than that taken for the food materials were labelled by the pheromone in the previous study (24 h). Secondly, oviposition was unlikely to occur on the odour sources without food materials. Nevertheless, the results of current study may imply the ecological consequence in group level. However, the results clearly showed that preference of group of flies was shifted to the environment on which they had been reared due to the sleep deprivation with night-time disturbance. In order to investigate behavioural changes at the level of individual flies and the physiological and cognitive processes involved, test assays of olfactory responses using individual flies are needed.

A future challenge is to find the neural basis for the preference for novel stimuli in *Drosophila* and its commonality with that in humans, which will provide insights into the development of treatments for neural disorders through sleep interventions. Studies have also shown that orthologous genes are activated during sleep in mammals and fruit flies^8,10^. Clarification of the biological basis concerning the function of sleep in *Drosophila* can provide an approach for research on the prevention or treatment of human neural disorders through sleep interventions. For example, histamine antagonists are used as hypnotics in humans. Flies given hydroxyzine, an antagonist of the H1 histamine receptor, showed increased rest and reduced latency^10^. Previous research showed that mutant mice lacking histamine H1 receptors demonstrated decreased exploratory behaviour in new environments^51^. Thus, histamine H1 receptors are a common biological basis that could be investigated to better understand the relationship between sleep and novelty-seeking behaviours using *Drosophila*. Moreover, a decline in novelty-seeking behaviour is also found in neural disorders, such as Alzheimer’s disease^52^. The early symptoms of Alzheimer’s disease are mainly memory problems, including recognition of novel objects and events^53^. Cognitive impairment can lead to difficulty in daily activities and major declines in patient quality of life. These symptoms of Alzheimer’s disease are accelerated by sleep loss through the accumulation of amyloid-β peptide (Aβ) in the patient’s brain, and its toxicity is reversed by sleep enhancement^54,55^. In studies using *Drosophila* mutants, sleep enhancement reversed memory impairment by abnormal processing and accumulation of Aβ and cAMP signalling deficits in clock neurons, which play important roles in sleep and memory consolidation in humans^56^. The relationship between these neural bases in *Drosophila* and exploration for novel odours found in the present study needs to be investigated.

## Methods

### Animals

*Drosophila melanogaster* flies were collected from fields near Shizuoka University and reared on standard cornmeal agar medium^57^ at 25 °C and 60–70% relative humidity under a 14 L:10 D photocycle (lights on at zeitgeber time 0 [ZT0] = 05:00) in a constantly ventilated environment. For the treatment of the rearing environment, medium without propionic acid was used, and 20% (w/v) apple cider vinegar (Jun-Ringo-Su; Mizkan, Japan) or 10% (w/v) broth (bonito soup stock) (Fuumi-Dashi; Yamasa, Japan) was supplemented (percentages represent final concentrations; these are referred to as ACV medium and broth medium, respectively). To prepare these media, solutions of ACV or broth were sterilized with a syringe filter (0.22 μm) and supplied to the medium while cooling. For behavioural experiments, adult flies of both sexes were used 3–4 days after hatching.

### Sleep deprivation

Sleep deprivation was conducted by periodic physical stimulation with rotation using a custom-made centrifuge device (Fig. 1a). In the device, the centrifuge tubes housing flies were attached to a horizontal fibreboard disk (30 cm diameter) so that the light was not blocked and their arrangement was symmetrical. The rotation speed was approximately 500 rpm. Previous literatures demonstrated that sleep deprivation achieved by displacing flies to the bottom of vials with tapping or dropping of vials^16,58^. In our current study, we observed all flies were displaced to the bottom of the centrifuge tubes after the rotation treatment. The lid of the tube had tiny holes for ventilation, and the bottom of the tube was filled with moistened cotton to prevent flies from becoming too dry or being injured from excessive pressure. A digital timer relay (TB201K; Panasonic, Osaka, Japan) was used to turn the machine on and off every minute for 8 h. Flies were treated as follows 12 h before the start of the experiments. “No rotation flies” were kept in centrifuge tubes with moistened cotton wool and experienced starvation from ZT14 to ZT2. Night-time and daytime rotated flies were kept in centrifuge tubes with moistened cotton wool and rotated for 8 h at different time schedules with 12 h of starvation, as night-time rotated flies experienced starvation from ZT14 to ZT2 and rotation from ZT15 to ZT23, and daytime rotated flies experienced starvation from ZT2 to ZT14 and rotation from ZT3 to ZT11. At the start of the experiment, all treated flies were placed in their respective olfactory test apparatus using an insect suction tube as a group of 50 flies.

### Locomotor activity

Flies with night-time rotation or those without rotation were individually introduced into a 5 × 65 mm glass tube with an infrared photo reflector (RPR-220, Rohm Co. Ltd., Kyoto, Japan) attached on its centre. One of the ends of the tube was plugged with standard cornmeal agar medium (covered with Parafilm), and the other side was plugged with cotton wool. Sensor reading was recorded every 100 milli seconds using an Arduino UNO R3 microcontroller^59^ and computer program built in Processing (version 3.5.4)^60^. Crossing of the fly was determined using Microsoft Excel. When the time of intervals between crossings was longer than 5 min^33,34^, it was regarded as sleep. The monitoring was started at ZT1 and continued until two photocycles ended. During the preparation for the monitoring, tubes housing flies were constantly tapped to prevent them from sleeping.

### Olfactory response (trap assay)

Olfactory preference was determined based on the methods of a previous study with modifications^23^. An olfactory test apparatus (Fig. 2) was fabricated from a 500 ml plastic container (Fig. 2a). The ceiling of the screw cap of the apparatus had an opening (8 mm diameter, Fig. 2b) for the rapid transfer of flies from the insect suction tube and 50 tiny holes for ventilation (Fig. 2c) that were too small for the flies to pass through. Two glass vials (Fig. 2d) were placed in the apparatus filled with 10 ml odour solutions (20% apple cider vinegar (Jun-Ringo-Su; Mizkan, Japan), 10% broth (bonito soup stock) (Fuumi-Dashi; Yamasa, Japan), or water) (Fig. 2e). The concentrations of solutions were determined based on the literature^22^ and preliminary experiments in which they are sufficient for attraction of flies. A hole (2 mm in diameter) was drilled in the lid of the vial cap to provide a path for the flies, and a tube made of a pipette tip was attached to the hole to prevent the flies from escaping (Fig. 2f). In the preliminary observation, 91.25 percent of trapped flies were remained in the trap vials within 24 h (tested with 240 flies divided in 12 vials). Most of flies shows the first choice with olfaction in this assay.

Flies starved for 12 h (including 8 h of periodic rotations in night-time/daytime rotation treatments) were released into the olfactory test apparatus using an insect suction tube. Fifty flies were transferred from the rearing tubes to a centrifuge tube and then to a test apparatus. If there were any dead flies immediately after release, the number was recorded. The apparatus was placed under the same temperature and humidity conditions as the rearing tubes. Flies were allowed to choose two odour source vials for 16 h. After 16 h, the number of flies in each odour source vial and flies not in either vial were recorded. In all experiments, 10 replicates were conducted. The order of each experiment and the positions of the odour source vials (left and right) in each test apparatus were randomly determined using random numbers generated by a computer program. Flies were subjected to one of the experiments only once.

The treatments of flies and odour sources used in each experiment were as follows.

Experiment 1 Preference for Apple cider vinegar (ACV) or broth: Flies with different rotation schedules (no rotation, night-time rotation, daytime rotation); 20% ACV vs 10% broth.

Experiment 2 Response rate to each odour source: Flies with different rotation schedules (no rotation, night-time rotation, daytime rotation); 20% ACV or 10% broth vs water.

Experiment 3 Effect of the rearing environment on the preference: Flies from different media (ACV medium or broth medium) with different rotation schedules (no rotation or night-time rotation); 20% ACV vs 10% broth.

### Statistical analysis

Statistical analysis was conducted using R (version 4.0.0) (R Core Team, 2020). In activity monitoring, activity of flies was analysed using generalized linear mixed models (GLMM) with Poisson distribution and log link function (glmmML function in glmmML package). The total time of sleep in the day was used as the response variable. The rotation treatments (night-time rotation or no rotation) and monitoring day (first or second) were used as the explanatory variables. Replicate (fly individual) was used as a random effect. In olfactory tests (experiment 1–3), the responses of flies were analysed using GLMM with binomial distribution and logit-link function (glmmML function in glmmML package). In experiments 1 and 3, the objective of the analysis was to test the preference between two different odours; i.e. our interest was in the distribution of flies between the two traps. Therefore, in experiments 1 and 3, the ratio of the number of flies trapped in each odour source vial was used as the response variable. In experiment 2, the objective of the analysis was to break down the preference of flies into the response to each single odour; i.e. our interest was in the number of flies in the trap with odour within the total number of flies subjected to the choice. Therefore, in experiment 2, the ratio of the number of flies trapped in the odour source vial to the number of live flies was used as the response variable. In experiments 1 and 2, only rotation (no rotation, night-time rotation, daytime rotation) was considered an explanatory variable. In experiment 3, rotation condition (no rotation, night-time rotation) and rearing environment (ACV medium, broth medium) were used as explanatory variables. In all cases of experiment 1–3, the replicate (test apparatus) was used as a random effect. Effects of the treatments were determined based on likelihood ratio tests using a parametric bootstrap simulation with 1000 replicates. The pairwise comparisons between the results of different treatments within the explanatory variable were performed using GLMM with the data of the two relevant treatments. The Holm-Bonferroni method was used to correct the *p*-values in multiple comparisons. For data visualization of the preference tests in experiment 1 and 3, the following preference index was used:

$$Preference \,Index = \frac{ACV\, choice - broth \,choice}{{total \,choice}}.$$

Using this index, preference was shown in the range between 1 (absolute preference for ACV) and − 1 (absolute preference for broth) with 0 as a neutral preference.

## Supplementary Information

Supplementary Information.
